# Onchocerciasis Prevalence among Persons with Epilepsy in an Onchocerciasis Hypo-Endemic Area in the Democratic Republic of Congo: A Cross-Sectional Study

**DOI:** 10.3390/pathogens10040389

**Published:** 2021-03-24

**Authors:** An Hotterbeekx, Kristien Verdonck, Deby Mukendi, Jean-Roger Lilo-Kalo, Pascal Lutumba, Marleen Boelaert, Liselotte Hardy, Barbara Barbé, Jan Jacobs, Emmanuel Bottieau, Robert Colebunders

**Affiliations:** 1 Global Health Institute, University of Antwerp, 2100 Antwerp, Belgium; robert.colebunders@uantwerpen.be; 2 Department of Public Health, Institute of Tropical Medicine, 2100 Antwerp, Belgium; tverdonck@itg.be (K.V.); lhardy@itg.be (L.H.); 3 Institut National de Recherche Biomédicale, Av. De la Démocratie N°5345, 1197 Kinshasa, Democratic Republic of the Congo; debymukendi@gmail.com (D.M.); lilo.kalo@yahoo.fr (J.-R.L.-K.); pascal_lutumba@yahoo.fr (P.L.); 4 Centre Neuro-Psycho Pathologique, Université de Kinshasa, 7948 Kinshasa, Democratic Republic of the Congo; 5 Department of Clinical Sciences, Institute of Tropical Medicine, 2100 Antwerp, Belgium; bbarbe@itg.be (B.B.); jjacobs@itg.be (J.J.); EBottieau@itg.be (E.B.); 6 Department of Microbiology and Immunology, Catholic University Leuven, 3000 Leuven, Belgium

**Keywords:** onchocerciasis, *Onchocerca volvulus*, *Taenia solium*, antibodies, epilepsy, cross-sectional study, prevalence, Democratic Republic of Congo

## Abstract

A high epilepsy prevalence has been reported in onchocerciasis meso- and hyper-endemic regions in sub-Saharan Africa, including in the Democratic Republic of Congo (DRC). We investigated whether onchocerciasis-associated epilepsy can also be suspected in onchocerciasis hypo-endemic regions. Stored serum samples from 342 patients admitted with recent onset neurological symptoms admitted to Mosango general hospital, in the Kwilu province, DRC, between 2012 and 2015 were screened for onchocerciasis (OV16) antibodies by ELISA and *Taenia solium* antigen (using an in-house B158/B60 antigen test). Eighty-one (23.7%; 95% CI 19.5–28.5%) of these samples were positive for OV16 antibodies and 43/340 (12.6%; 95% CI 9.5–16.6%) were positive for *T. solium* antigen. Of the 58 persons clinically diagnosed with late onset epilepsy of unknown etiology, 19 (32.8%) were OV16 positive and nine (16%) *T. solium* antigen positive. In total, 16 persons with epilepsy were OV16 positive and *T. solium* negative, of whom 12 (75%) were between the ages seven to 31 years old, an age rage in which onchocerciasis-associated epilepsy is observed. Our study suggests that in onchocerciasis hypo-endemic areas, in *T. solium* antigen negative persons with epilepsy, onchocerciasis should be considered as a potential trigger of epilepsy.

## 1. Introduction

Neurological disorders in low and middle income countries have a variety of causes, including various infectious diseases [1,2]. Many of these diseases, such as cerebral malaria, bacterial meningitis, and human African trypanosomiasis (HAT) are treatable when the correct diagnosis is made in time [1,2]. The latter remains challenging in the rural settings where these diseases are most prevalent, especially in the early stages where most people have nonspecific symptoms and advanced diagnostic tools are absent. This leads to delay of diagnosis and consequently a larger disease burden in the affected communities.

A study was conducted between 2012 and 2015 to improve field diagnosis of common and severe neglected neurological diseases in Mosango general referral hospital in Kwilu province, Democratic Republic of Congo (DRC) [3]. In this study, patients above five years old who were admitted to the hospital with recent-onset neurological symptoms were recruited [3]. These patients all had a normal development without neurological symptoms before the age of five years. In 18% of the patients, the neurological symptoms could be attributed to common infections of the central nervous system, such as meningitis, HIV and related infections, and HAT [3]. However, many of them ended up with a syndromic diagnosis (without etiological agent), mainly epilepsy. In a post-hoc study using the stored samples of these neurological patients, we determined the presence of *Taenia solium* antigens and found 12.6% positivity in the whole neurological cohort and 16% positivity in the subset with clinical diagnosis of epilepsy [4].

High epilepsy prevalence has been reported in onchocerciasis meso- and hyper-endemic regions (i.e., regions where >20% of adults present onchocerciasis nodules [5,6,7,8]) including in the DRC [9,10,11,12,13,14]. In such regions, a form of epilepsy, called onchocerciasis-associated epilepsy, has been described. This form of epilepsy is characterized by the following criteria [15]: (1) the person has lived in an onchocerciasis endemic region for at least three years (2) onset of seizures occurred between 3–18 years of age; (3) there is a high prevalence of epilepsy in the village, and there are several families with more than one child with epilepsy in this village; (4) there is no obvious cause of epilepsy (for example perinatal trauma, recent head trauma, cerebral malaria, encephalitis, or neurocysticercosis); (5) prior to the onset of epilepsy, the psychomotor development of the child was normal; and (6) the person presents onchocerciasis antibodies and/or microfilariae in skin snips.

What we do not know is whether certain persons with epilepsy in onchocerciasis hypo-endemic regions may have onchocerciasis-associated epilepsy. We were interested to screen persons with neurological conditions including epilepsy in an onchocerciasis hypo-endemic area for the presence of *Onchocerca volvulus* antibodies. Initially, *O. volvulus* was not considered as a potential causative agent of neurological symptoms in the study in Mosango. We therefore set out to determine the prevalence of *O. volvulus* antibodies in the Mosango neurological study population in general, and in patients with epilepsy without evidence of *T. solium* infection in particular. We hypothesized that a limited number of persons with epilepsy would suffer from onchocerciasis-associated epilepsy.

## 2. Materials and Methods

### 2.1. Study Design

#### 2.1.1. Study Population

This study is part of the project “Better Diagnosis for Infectious Diseases” (NIDIAG; www.nidiag.eu, accessed on 19 March 2021). Consecutive patients with recent-onset neurological disorders admitted to Mosango general referral hospital in Kwilu province, DRC, were recruited between 2012 and 2015. The study area is classified as hypo-endemic according to rapid epidemiological mapping of onocherciasis (REMO). Detailed description of the patient population and inclusion criteria are published elsewhere [3]. All eligible patients were older than five years, with at least one of the following symptoms: (1) altered state of consciousness, (2) changes in sleep pattern, (3) cognitive decline, (4) changes in personality/behavior, (5) recent epileptic seizure (within less than two weeks), (6) recent, severe and progressive headache, (7) meningism, (8) new onset cranial nerve lesion(s), (9) new onset sensory-motor focal deficits, and (10) new onset gait/walking disorders. These symptoms had to be either of recent onset or ongoing for a longer time but still present on admission. Patients younger than five years or with neurological symptoms due to recent trauma or a past neurological event were not eligible for the study [3].

#### 2.1.2. Diagnosis of Epilepsy

Symptoms and diagnosis of epilepsy were registered in the following ways: (1) as epileptic seizure regardless the etiology as a motive for inclusion in the study or (2) as late-onset epilepsy of unknown etiology (using the 2014 ILAE definition of epilepsy [16] and after ruling out a set of infectious diseases). The conditions for which the study participants were systematically tested were: (1) second-stage HAT, (2) cerebral malaria, (3) bacterial meningitis and unspecified meningoencephalitis, (4) tuberculosis of the CNS, (5) neurosyphilis, and (6) HIV-related neurological disorders.

#### 2.1.3. Laboratory Tests for *Onchocerca volvulus* and *Taenia solium* Infection

Cryopreserved serum collected during the clinical study was retrospectively screened for OV16 IgG4 antibodies by enzyme-linked immunosorbent assay (ELISA) with Horseradish peroxidase (HRP) as described earlier [17]. Briefly, plates were coated with OV16 antigen (Abcam, Cambridge, UK) overnight and washed three times with washing buffer (Phosphate buffered saline with 0.5% Tween 20). Plates were blocked with SuperBlock Blocking buffer (Invitrogen, Carlsbad, CA, USA) for 30 min and washed three times. Samples were diluted 1:200 and incubated at room temperature for 1 h, followed by five washing steps. HRP-conjugated anti-human IgG antibodies (Abcam) were used as detection antibody, diluted 1:10,000 and incubated for 1 h. After five washing steps, one-step Ultra TMB substrate solution (ThermoFisher Scientific, Waltham, MA, USA) was added, and the reaction was stopped by adding 1N HCl after 10 min. The absorbance was measured at an optical density of 450 nm. *T. solium* antigens were also measured on collected sera by an in-house B158/B60 antigen ELISA as described before [18]. This test has a sensitivity of 90% to 100% to detect current cysticercosis and a specificity of 83% to 98% [19].

### 2.2. Data Analysis

We first determined the frequency of OV16 antibody positivity among all patients with neurological disorders and compared demographic and clinical characteristics of OV16-seropositive and OV16-seronegative patients. We then described the age distribution in OV16-seropositive and *T. solium* antigen-positive patients. Next, we focused on the subgroup of patients clinically diagnosed with epilepsy and described the characteristics of epilepsy patients with positive versus negative tests for *O. volvulus* (OV16 antibody test) and *T. solium* (in-house B158/B60 antigen test). We used the chi-squared test with Yates’ continuity correction, the Fisher exact test, and the Wilcoxon rank sum test to assess statistical significance, taking an alpha level of 0.05. We used Microsoft Excel for data processing and R for statistical analyses.

## 3. Results

### 3.1. All Patients with Neurological Disorders

**Figure 1:**
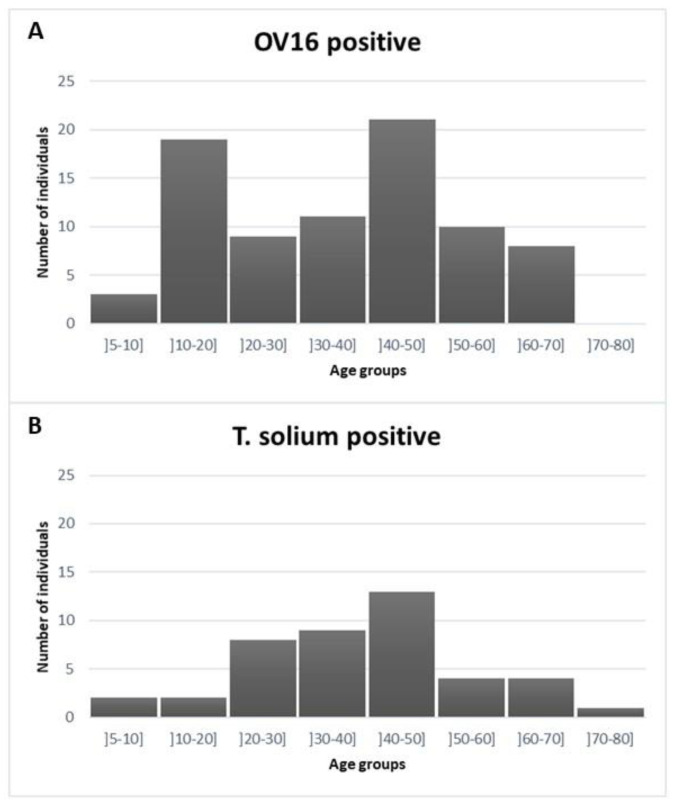
Distribution of age in OV16-seropositive (panel (**A**)) and *T. solium* antigen-positive patients (panel (**B**)).

Serum samples from 342 out of 351 (97%) patients with neurological disorders enrolled in the NIDIAG study were available for OV16 testing, while 340 had been tested previously for *T. solium* testing. Eighty-one (23.7%; 95% CI 19.5–28.5%) of these samples were positive for the presence of OV16 antibodies and 43/340 (12.6%; 95% CI 9.3–16.7%) were positive for *T. solium* antigen. The median age of the OV16-seropositive patients was 40 years (interquartile range 20 to 50) and that of the OV16-seronegative patients 41 years (interquartile range 25 to 54; *p* = 0.2). There were three OV16-seropositive children under 10 years old (one child of six and two children of seven years old) and the prevalence of OV16 positivity among the 10–20 year old age group was 33% (Figure 1). The median age of the *T. solium* antigen-positive patients was 42 years (interquartile range 31 to 50). The prevalence of antigen positivity was 4/70 (5.7) % up to the age of 20 years.

There were no statistically significant differences in demographic and clinical characteristics when we compared 81 OV16-seropositive with 261 OV16-seronegative patients (Table 1).

Twenty-four percent of men and 23% of women were OV-16 seropositive (*p* = 0.9). Skin and soft tissue symptoms (n = 7) and itching (n = 2) were uncommon in the study population; all participants with these symptoms were OV16 seronegative. The majority of patients (n = 227; 66%) came from the Mosango health zone, and this proportion was similar among OV16-seropositive (65%) and OV16-seronegative (67%) patients.

### 3.2. Persons with Epilepsy

The study population included 58 patients with a final diagnosis of epilepsy. Nineteen of them (32.8%) were OV16 seropositive. In 57 of these 58 epilepsy patients, results of the antigen test for *T. solium* were also available, and nine out of 57 (16%) were positive. Detailed results of the two tests are given in Table 2.

Taking all diagnostic test results together, there were 16 persons with epilepsy (28%) with a positive serology for onchocerciasis and a negative antigen test results for cysticercosis. Other infectious diseases (HIV, tuberculosis, malaria, second-stage human African trypanosomiasis, bacterial meningitis, and neurosyphilis) were ruled out in these patients. The characteristics of these 16 patients, for whom onchocerciasis could be the trigger of epilepsy, are summarized in Table 3. They all had a recent epileptic seizure (within less than two weeks) when they were enrolled. The median age of the 16 persons with epilepsy that were OV16 positive only was 22 years (interquartile range 18 to 34 years), and that of the six only *T. solium* antigen positive epilepsy patients was 33 years (interquartile range 27 to 35 years, *p*-value 0.18).

## 4. Discussion

This study among patients with neurological disorders enrolled at the Mosango hospital, a rural hospital located in an onchocerciasis hypo-endemic area of the DRC, revealed that 23.7% were OV16 ELISA test positive, with no other evidence of infectious etiologies including cysticercosis. As the OV16 ELISA test has a high specificity for the presence of *O. volvulus* antibodies [20], this means these individuals had been exposed to this infection. Moreover, the finding of three OV16-seropositive children under 10 years old suggests that there was relatively recent ongoing onchocerciasis transmission in the area where these children originated from (Mosango and Masimanimba). A slightly higher percentage (32.8%) of the people with epilepsy were positive for onchocerciasis, but this difference was not significant. Among the 57 persons with epilepsy, 16 (28%) presented *O. volvulus* antibodies only. No information was available about the age of onset of the epilepsy, but because 12 (75%) of the 16 persons with epilepsy were between the ages 7–31 years old, it is possible that some of them had onchocerciasis-associated epilepsy. The mean age of onset of onchocerciasis-associated epilepsy is between 10 and 12 years, and nearly all affected persons die before the age of 35 [7]. Severe progressive headache is generally not a symptom of onchocerciasis-associated epilepsy. Therefore, in patient 8 and 9 and in particular patient 9, who presented meningism, most likely the epilepsy was not triggered by onchocerciasis. Certainly, the epilepsy of the 60 year old person from Kinshasa was not related to onchocerciasis. It is interesting to note that the OV16 seropositivity started at an earlier age than *T. solium* antigen positivity. This may also explain that onchocerciasis associated epilepsy is generally observed at an earlier age than epilepsy caused by neurocysticercosis.

Our study has several limitations. First, no skin snips were taken to determine active *O. volvulus* infection and infection load. A positive OV16 antibody test is only an indication of exposure and does not provide information on current infection. Furthermore, no information on past ivermectin use was collected. Second, data on the area of residence and duration of residence in the area were unknown, as only the location from where the patient was admitted to the hospital was collected. Therefore, it is not certain which patients were residing or had in the past resided in regions meso- or hyper-endemic for onchocerciasis. Finally, no brain imaging was performed in the persons with epilepsy

Despite these limitations, our study suggests that there may be *O volvulus* transmission in the broader Mosango area and that some of the epilepsy was induced by onchocerciasis. It has been suggested that neurocysticercosis is the most important parasitic cause of epilepsy in sub-Saharan Africa. However, in Mosango, more persons with epilepsy tested positive for the presence of *O. volvulus* antibodies (28%) than *T. solium* antigen (11%) only. Our study suggest that in onchocerciasis hypo-endemic areas in *T. solium* antigen negative persons with epilepsy, onchocerciasis should be considered as a potential trigger of epilepsy. Moreover, if our OV16 prevalence data are confirmed in a community based study, the Congolese onchocerciasis elimination program should consider including the Mosango area for onchocerciasis elimination mapping [21].

## Figures and Tables

**Table 1:** Number and proportion of patients with positive serology for *Onchocerca* stratified by demographic characteristics, clinical symptoms and signs, and final diagnosis.

Patient Characteristic	Number with Positive Serology	Number Tested	Proportion with Positive Serology	Risk Ratio (95% CI)	*p*-Value *
Sex					
Women	43	186	23.1%	1	0.89
Men	38	156	24.4%	1.05 (0.72, 1.54)	
Age category					
<10 years	3	15	20.0%	1	1.00
≥10 years	78	327	23.9%	1.19 (0.43, 3.34)	
Neurological symptoms/signs at presentation					
Epileptic seizure	27	87	31.0%	1.47 (0.99, 2.17)	0.09 **
Gait/walking disorders	27	95	28.4%	1.30 (0.87, 1.93)	0.26
Focal sensory-motor deficit	20	73	27.4%	1.21 (0.78, 1.86)	0.49
Behaviour disturbance	17	63	27.0%	1.18 (0.74, 1.86)	0.60
Altered state of consciousness	13	53	24.5%	1.04 (0.62, 1.75)	1.00
Change in sleep pattern	10	47	21.3%	0.88 (0.49, 1.59)	0.82
Cranial nerve lesion	4	19	21.1%	0.88 (0.36, 2.16)	1.00
Severe headache	32	156	20.5%	0.78 (0.53, 1.15)	0.26
Cognitive decline	3	18	16.7%	0.69 (0.24, 1.98)	0.58
Signs of meningism	16	108	14.8%	0.53 (0.32, 0.88)	0.01
Skin or soft tissue symptoms	0	7	0.0%		0.21
Itching	0	2	0.0%		1.00
Localised adenopathy	6	19	31.6%	1.36 (0.68, 2.71)	0.41
Final diagnosis of late onset epilepsy of unknown etiology	19	58	32.8%	1.50 (0.98, 2.31)	0.11 **
Total	81	342	23.7%		

95% CI: 95% confidence interval; * Chi-squared test with Yates’ continuity correction or Fisher exact test (if expected cell count < 5). ** Excluding all *T. solium* antigen positive-individuals from the analysis, the *p*-value for epileptic seizures becomes 0.046 and for final diagnosis of late onset epilepsy of unknown origin 0.07 (Supplementary Table).

**Table 2:** Test results for *Onchocerca volvulus* (OV16 antibody test) and for *Taenia solium* (in-house B158/B60 antigen test) in 57 patients with a diagnosis of epilepsy, stratified by sex and age.

Characteristic	Both Tests Positive n (%)	Only *Onchocerca* Antibody Test Positive n (%)	Only *Taenia* Antigen Test Positive n (%)	Both Tests Negative n (%)	Total
Sex					
Women	0 (0)	9 (26)	3 (9)	22 (65)	34
Men	3 (13)	7 (30)	3 (13)	10 (43)	23
Age					
<20 years	1 (5)	6 (30)	1 (5)	12 (60)	20
≥20 years	2 (5)	10 (27)	5 (14)	20 (54)	37
Total	3 (5)	16 (28)	6 (11)	32 (56)	57

**Table 3:** Characteristics of 16 patients with epilepsy, a positive test result for *Onchocerca volvulus* (OV16 antibody test) and a negative test result for *Taenia solium* antigen (in-house B158/B60 antigen test).

Nr	Sex	Complaints at Enrolment Apart from Seizures	Age at Enrolment	Age at Epilepsy onset	Place of Residence	Type of Seizures
1	F	None	7	7	Mosango	generalised
2	F	None	12	12	Mosango	generalised
3	M	Changes in personality/behavior, recent, severe, and progressive headache	18	18	Mosango	generalised
4	F	None	18	18	Mosango	generalised
5	F	None	18	18	Mosango	generalised
6	M	Altered state of consciousness	19	19	Mosango	localised
7	F	None	20	20	Mosango	generalised
8	F	Recent, severe, and progressive headache	20	21	Masimanimba	generalised
9	F	Recent, severe, and progressive headache, meningism	24	28	Mosango	generalised
10	F	None	25	25	Mosango	generalised
11	M	None	25	25	Masimanimba	generalised
12	M	Altered state of consciousness	31	31	Masamuna	generalised
13	M	Changes in personality/behaviour, recent, severe, and progressive headache	42	42	Masimanimba	generalised
14	M	Recent, severe, and progressive headache	44	44	Mosango	generalised
15	F	Recent, severe, and progressive headache	47	46	Masimanimba	generalised
16	M	Changes in sleep pattern, cognitive decline, changes in personality/behavior	60	60	Kinshasa	generalised

F = Female, M = Male.

## Data Availability

The datasets generated during the current study are available from the corresponding authors on reasonable request.

## Supplementary Materials

The following are available online at https://www.mdpi.com/2076-0817/10/4/389/s1. Table S1: In a subset of 290 patients with negative antigen test results for *Taenia solium*: number and proportion of patients with positive serology for *Onchocerca*, stratified by demographic characteristics, clinical symptoms and signs, and final diagnosis.
