# Supplementary material for: Onchocerciasis Prevalence among Persons with Epilepsy in an Onchocerciasis Hypo-Endemic Area in the Democratic Republic of Congo: A Cross-Sectional Study
Source: Pathogens. 2021 Mar 24;10(4):389. doi: 10.3390/pathogens10040389 (PMC8063918; doi:10.3390/pathogens10040389)
Supplement: Supplementary file 1 [file pathogens-10-00389-s001.pdf]

Supplementary table. In a subset of 290 patients with negative antigen test results for *Taenia solium*: number and proportion of patients with positive serology for *Onchocerca*, stratified by demographic characteristics, clinical symptoms and signs, and final diagnosis,.

Patient characteristic	Number with positive serology	Number tested	Proportion with positive serology	P-value*
Sex				
Men	29	130	22.3%	1.00
Women	36	160	22.5%	
Age category				
<10 years	2	14	14.3%	0.74
≥ 10 years	63	276	22.8%	
Neurological symptoms/signs at presentation				
Epileptic seizure	23	73	31.5%	0.046
Gait/walking disorders	21	84	25.0%	
Focal sensory-motor deficit	16	63	25.4%	
Behaviour disturbance	13	53	24.5%	
Altered state of consciousness	10	44	22.7%	
Change in sleep pattern	9	41	22.0%	
Cranial nerve lesion	4	18	22.2%	
Severe headache	28	133	21.1%	
Cognitive decline	1	12	8.3%	
Signs of meningism	12	91	13.2%	
Skin or soft tissue symptoms	0	5	0.0%	
Itching	0	2	0.0%	
Localised adenopathy	4	16	25.0%	
Final diagnosis of late onset epilepsy of unknown etiology	16	48	33.3%	0.07
Total	65	290	22.4%	
